# Case report: Brain metastasis necrosis with immune checkpoint inhibitors plus chemotherapy for advanced non-small cell lung cancer

**DOI:** 10.3389/fimmu.2022.1064596

**Published:** 2022-12-01

**Authors:** Lishui Niu, Xiang Li, Li Meng, Yingying Zhang, Xin Wan, Di Jing, Qin Zhou, Rongrong Zhou

**Affiliations:** ^1^ Department of Oncology, Xiangya Hospital, Central South University, Changsha, China; ^2^ Department of Pathology, Xiangya Hospital, Central South University, Changsha, China; ^3^ Department of Radiology, Xiangya Hospital, Central South University, Changsha, China; ^4^ Department of Neurosurgery, Xiangya Hospital, Central South University, Changsha, China; ^5^ Xiangya Lung Cancer Center, Xiangya Hospital, Central South University, Changsha, China; ^6^ National Clinical Research Center for Geriatric Disorders, Xiangya Hospital, Central South University, Changsha, China

**Keywords:** case report, lung cancer, brain necrosis, brain metastasis, immune checkpoint inhibitors

## Abstract

The emergence of immune checkpoint inhibitors (ICIs) has reshaped the landscape of advanced lung cancer treatment. The brain is the most common metastatic site for lung cancer. Whether conventional criteria can evaluate the intracranial response of ICIs remains unclear. Here, we report a well-documented case of intracranial necrosis confirmed by post-operative pathology after only one cycle of chemo-immunotherapy without any radiation therapy, which suggests that immunotherapy elicits strong anti-tumor responses for intracranial metastasis and promotes intracranial necrosis, resulting in a temporary increase in size of the target lesions. Still, the specific mechanisms and management strategies need to be further explored.

## Introduction

Non-small cell lung cancer (NSCLC) accounts for 85% of all lung cancers, with approximately 20% of patients presenting brain metastases (BM) at the time of initial diagnosis (1). Radiotherapy is currently the standard local treatment for BM. Due to the existence of the blood-brain barrier (BBB), most anti-tumor agents are difficult to penetrate into the intracranial metastasis sites (2). In recent years, the third-generation epidermal growth factor receptor tyrosine kinase inhibitors (EGFR-TKIs) have significantly prolonged the survival time and further improved the quality of life for NSCLC patients with BM who harbor sensitive EGFR mutations (3). However, treatment for driver-mutation-negative BM remains a big challenge. Most clinical trials permanently exclude patients with untreated or unstable intracranial metastases, and only 108 (17.5%) and 48 (7.9%) patients with brain metastasis were enrolled in KEYNOTE-189/KEYNOTE-407 trial, respectively, resulting in limited evidence supporting the efficacy of immune checkpoint inhibitors (ICIs) on BM lesions (4, 5). Even still, several subgroup analyses or small sample studies provided meaningful data, which suggested that ICIs could be active on BM (6). Here, we describe an NSCLC patient, with symptomatic brain metastasis, whose brain lesion was in complete response and replaced by necrosis after undergoing ICIs combined with chemotherapy without any radiation therapy.

## Presentation

**Figure 1:**
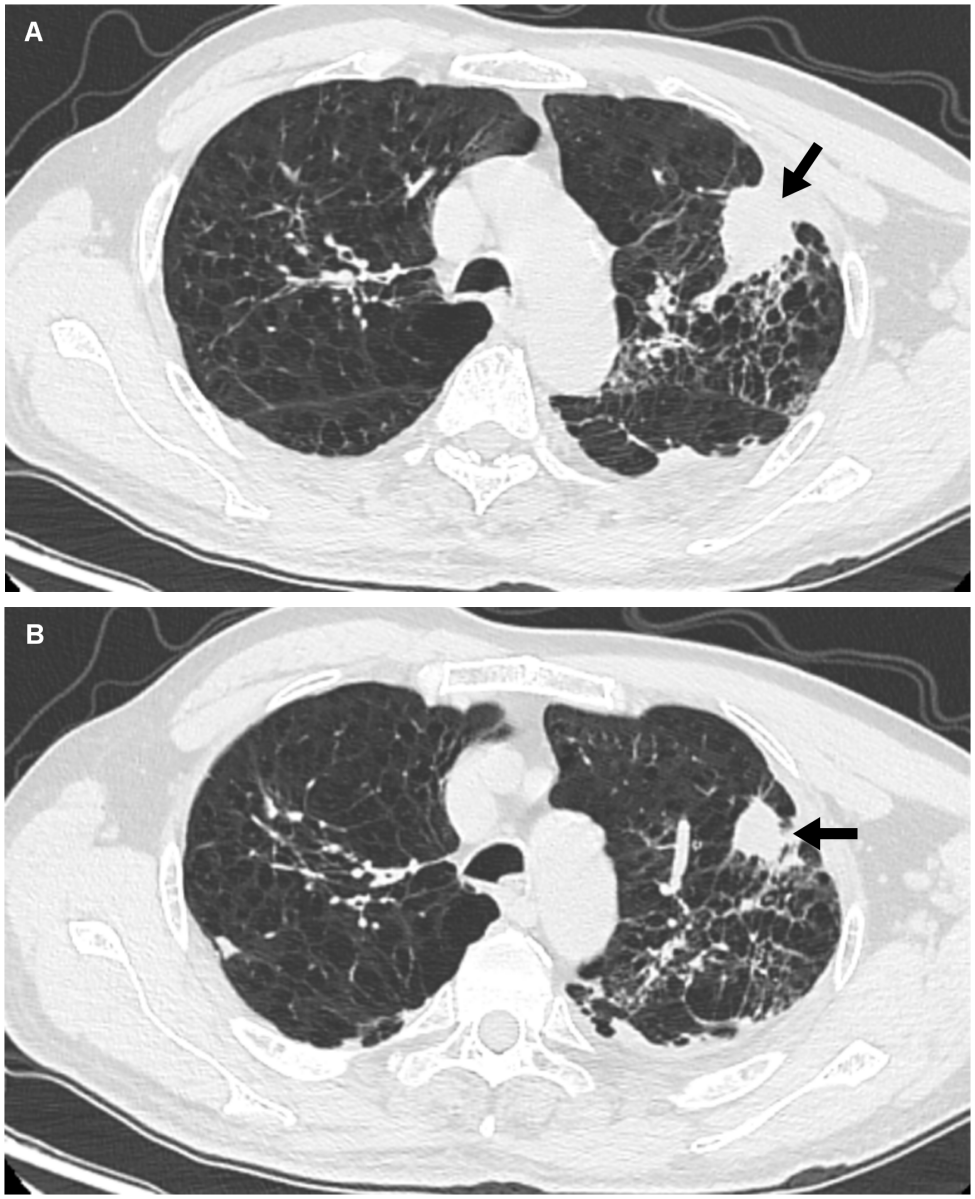
Findings of chest CT. **(A)** Image from chest CT shows a mass (arrow) in the apicoposterior segment of the left upper lung before ICIs therapy. **(B)** After one cycle ICIs treatment, the lung mass gradually shrank (arrow).

**Figure 2:**
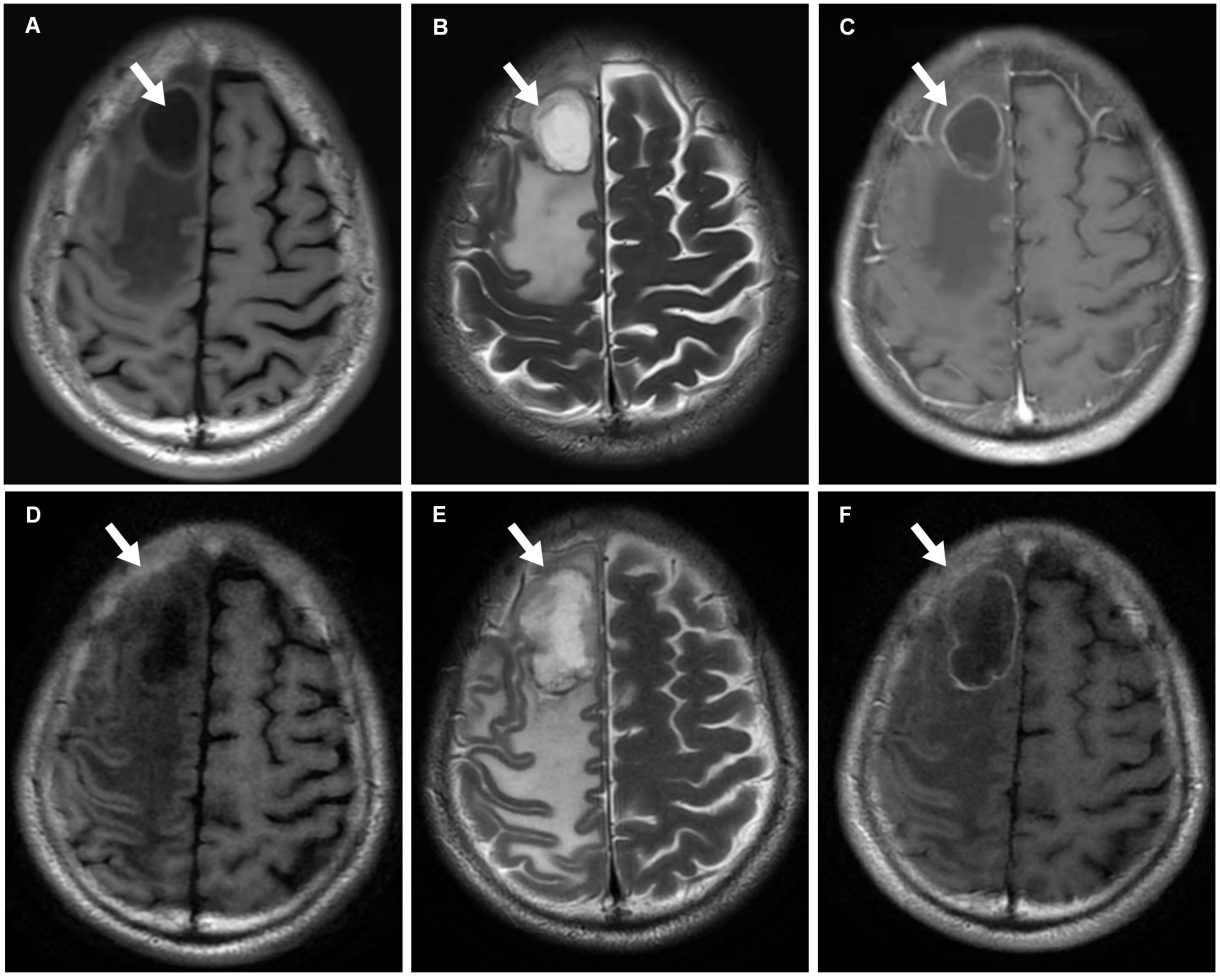
MRI scans. **(A–C)** In October 2021, T1-weighted (Panel **A**), T2-weighted (Panel **B**) and T1+C (Panel **C**) MRI scans reveal metastasis with cystic change and annular enhancement in the right frontal lobe (arrows). **(D–F)** After one cycle ICIs treatment, T1-weighted (Panel **D**), T2-weighted (Panel **E**) and T1+C (Panel **F**) MRI findings show that the tumor (arrows) increases.

**Figure 3:**
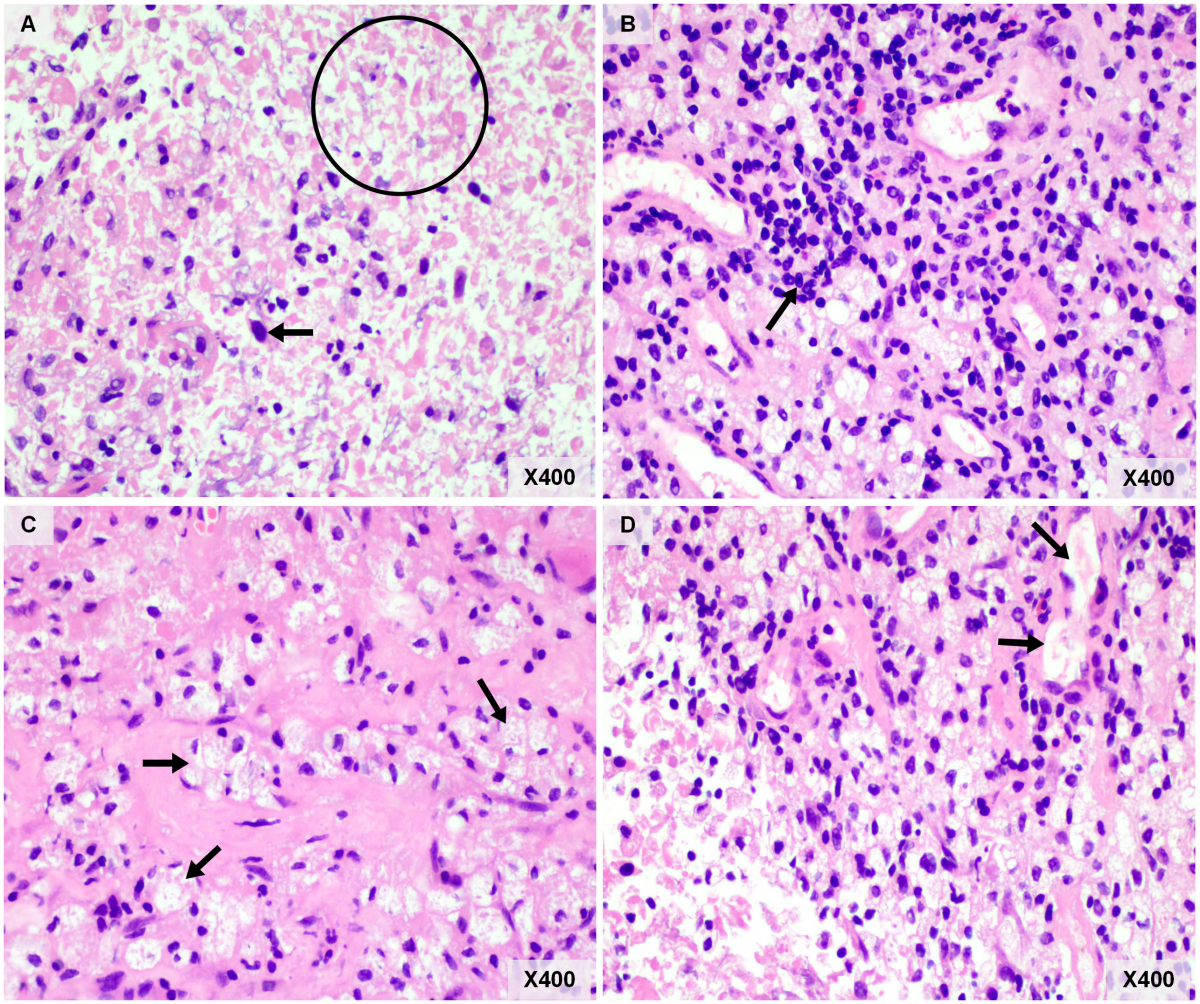
Pathological examination of resected brain metastasis. **(A)** Necrosis was common in brain metastasis (circle), and necrotic tumor cells (arrow) can be observed. **(B)** Lymphocytic infiltration (arrow). **(C)** There are numerous foam cells (arrows) were seen in the visual field. **(D)** The neovascularization was seen around the tumor bed (arrows). Original magnification: Panel **(A–D)**, hematoxylin and eosin, ×400.

An older patient with a history of 40 pack-years of smoking presented with blunted reactivity, mild consciousness disturbance, and left limb hemiplegia. A mass in the apicoposterior segment of the left upper lung (35mm*25mm) and a right frontal lobe cystic lesion with T1 and T2 hyperintensity and annular enhancement (29mm*22mm) were revealed by CT (**Figure 1**) and MRI (**Figure 2**), respectively. Pathological review of the needle biopsy demonstrated adenocarcinoma. Based on the results obtained above, the patient was then diagnosed with lung adenocarcinoma of stage IVa with BM (T2aN0M1b) in October 2021. Biopsy samples were sequenced using a 1021 genes panel (Geneplus, Beijing, China), demonstrating that tumor mutation burden (TMB) was 1.92Muts/Mb. No actionable driver gene mutation was detected. Immunohistochemical (IHC) analysis showed that tumor proportion score (TPS) was less than 1% (22C3 pharmDx assay on the Dako Autostainer Link 48 platform Carpinteria, Ca). The patient then received pembrolizumab (200mg, q3w, Merck & Co., Inc.) in combination with pemetrexed (500mg/kg, q3w, Eli Lilly & Co., Inc.) and carboplatin (AUC = 5, q3w, Qilu Inc.) as first-line therapy. Following the initial cycle of chemo-immunotherapy, the patient’s neurological symptoms were aggravated. The chest CT demonstrated that the lung mass had significantly decreased (**Figure 1**). The brain MRI detected a larger space-occupying lesion in the right frontal lobe with severer perifocal edema (**Figure 2**). The multi-disciplinary team (MDT) discussion suggested that surgical treatment could be performed. Subsequently, the patient received an excision of BM lesion, and necrosis was found in focal areas. Pathological findings revealed infiltration of lymphocytes and foam cells with necrosis. No tumor cell was observed (**Figure 3**). IHC analysis showed CK-Pan (-), EMA (-), and TTF-1 (-) in the necrotic areas. Given the absence of residual tumor cells in the resection margin, radiotherapy was not administered. After six cycles of chemo-immunotherapy, MDT recommended lung lesion surgery or radiotherapy. Based on the patient’s preference, radiotherapy was applied to the lung lesion. Now the patient’s condition is stable during pembrolizumab maintenance treatment.

## Discussion

This case report presents a rare case of BM necrosis after ICIs treatment combined with chemotherapy without any cranial radiation therapy. Tumor necrosis is always caused by tumor quick growth and treatment. In treatment-naive tumor, necrosis is characterized by its central location in the tumor nest and only part of tumor tissue develops necrosis. In this current study, the features of necrosis histology is inconsistent with treatment-naive tumor necrosis, so we think the necrosis of brain metastasis (BM) is caused by treatment (ICI or chemotherapy). Although previous studies have demonstrated that pemetrexed can penetrate the blood-tumor barrier (BTB), the concentration of pemetrexed is limited (7). The brain has a relatively suppressive immune tumor microenvironment (TME), which is characterized by decreased CD4+ helper T cells and CD8+ cytotoxic T cells infiltration, as well as low programmed cell death protein 1 (PD-L1) and TMB levels (8–10). Previous studies have reported that immunotherapy was able to induce necrosis of extracranial lesions with some unique histologic features, including dense tumor infiltrating lymphocytes with macrophages, tertiary lymphoid structures, proliferative fibrosis, and neovascularization (11, 12). Similar histopathological changes were also observed in this case, including massive tumor cell death, lymphoid infiltrates or aggregates, interstitial foamy macrophages and neovascularization. To date, the mechanism of BM necrosis induced by ICIs remains unclear because of the lack of case reports. We hypothesize that it may be related to the unique TME characteristics of BM and the potential role of ICIs in BM. The possible mechanisms of ICIs on intracranial lesions can be summarized as direct effects and indirect effects. It has been reported that brain metastases may disrupt the BBB so that ICIs and immune cells could directly traffick into the brain (13). We refer to these as “direct effects” (14). Alternatively, the anti-tumor effects of ICIs could also be relatively independent on direct contact with tumor cells. The preclinical study discovered that T-cell priming in the extracranial compartment was necessary for a successful immune response (15). A small amount of CD4+ T cells were reported to be present in a normal brain, and activated CD4+ T cells in the brain were able to increase the permeability of the BBB, which might allow circulating antibodies into the brain (14, 16). Lymphatic capillaries in the dura mater might also facilitate CNS antigen presentation in peripheral lymph nodes (17). Our case supports the hypothesis that BM necrosis could be related to the efficacy of ICIs treatment, which enhances immune cell infiltration and initiates penetration across the BBB.

We report, for the first time, a well-documented case of a patient with intracranial necrosis after only one cycle of chemo-immunotherapy treatment. Linlin Xiao et al. reported a patient with esophageal squamous cell carcinoma whose BM lesion had a complete response to ICIs and was replaced by a necrotic area after fifteen cycles of ICIs monotherapy (18). However, there was no pathological and perfusion imaging evidence supporting the diagnosis of necrosis. The clinical response pattern induced by ICIs differs from other treatment modalities. Pseudoprogression, dissociated response, and hyperprogression were described in previous studies (19, 20). It is still a big challenge to distinguish between BM progression and necrosis by routine imaging alone. Since tumor progression is closely associated with neovascularization, hyperperfusion signal in MRI could be effectively detected. Thus, the perfusion-weighted imaging (PWI) is generally used for differential diagnosis between tumor progression and necrosis. PWI has been used to assess tumor vascularity, including relative cerebral blood volume (rCBV), cerebral blood flow (CBF), capillary permeability (Ktrans), and mean transit time (MTT) (21). Besides, MR spectroscopy, positron emission tomography (PET), and histopathology also enable diagnostic discrimination (22). The accuracy of 6-[(18)F]-fluoro-L-3,4-dihydroxyphenylalanine (F-DOPA) PET for distinguishing tumor progression from radiation brain necrosis (RBN) of BM was even more than 90% (22). In our case, the initial diagnosis of intracranial lesion was considered to be tumor progression rather than necrosis based on MRI characteristics. Therefore, further advanced imaging examinations were not performed. However, the post-operative pathological results revealed widespread necrosis and no residual tumor cells in the resected specimen.

There could be some disagreements between radiographic and pathological evaluations, which may be frequently observed in perioperative immunotherapy. A recent study by Zhigang Liu et al. reported that three patients were confirmed to achieve pCR after surgery, while only one patient achieved radiographic PR according to the response evaluation criteria in solid tumors (RECIST) (23). Lee JM, et al. summarized the data of pathological and radiographic response of several neoadjuvant immunotherapy clinical trials of NSCLC, demonstrating large differences in results when the two methods of evaluation were compared (24). In our case, we found inconsistent response patterns of intracranial and extracranial lesions for immunotherapy. And there were obvious discordances between the results generated from MRI imaging and pathological review for intracranial metastasis, which emphasized the necessity of advanced imaging examination prior to local treatment (e.g., PWI). Atypical response patterns of ICIs should also be considered in clinical practice.

Currently, however, there is no evidence-based guideline yet for the diagnosis and treatment of immune-related intracranial necrosis. Clinically, radiation-induced brain necrosis is more common and there are various treatment strategies, including regular imaging follow-up (close observation for small and asymptomatic lesions), hyperbaric oxygen therapy (HBOT), medications, and surgery (25). Whether these strategies can be applied to immune-related intracranial necrosis still requires further investigation.

## Conclusion

We report a well-documented case of intracranial necrosis after only one cycle of ICIs treatment plus chemotherapy without any radiation therapy. Our case report provides direct evidence supporting the efficacy of anti-PD-1 therapy in NSCLC patients with brain metastases. Atypical response patterns of ICIs could be a major challenge for response evaluation. Comprehensive assessment for perfusion imaging, pathological evaluation, and clinical symptomatology could be essential to differentiate immune-related intracranial necrosis from tumor progression.

## Data availability statement

The original contributions presented in the study are included in the article/**Supplementary Material**. Further inquiries can be directed to the corresponding authors.

## Author contributions

RZ supervised the study. XL, LM, XW, and YZ collected data. LN performed the analysis and wrote paper. DJ and QZ provided substantial modification to the manuscript. All authors contributed to the article and approved the submitted version.

## Funding

This study was supported by the National Multidisciplinary Cooperative Diagnosis and Treatment Capacity Building Project for Major Diseases (Lung Cancer, grant number: z027002).

## Acknowledgments

We thank Dr. Chao Yuan for proofreading this article.

## Conflict of interest

The authors declare that the research was conducted in the absence of any commercial or financial relationships that could be construed as a potential conflict of interest.

## Publisher’s note

All claims expressed in this article are solely those of the authors and do not necessarily represent those of their affiliated organizations, or those of the publisher, the editors and the reviewers. Any product that may be evaluated in this article, or claim that may be made by its manufacturer, is not guaranteed or endorsed by the publisher.
