# Supplementary material for: Case report: Brain metastasis necrosis with immune checkpoint inhibitors plus chemotherapy for advanced non-small cell lung cancer
Source: Front Immunol. 2022 Dec 1;13:1064596. doi: 10.3389/fimmu.2022.1064596 (PMC9752014; doi:10.3389/fimmu.2022.1064596)
Supplement: Supplementary file 1 [file DataSheet_1.docx]

**Figure S1. Low magnification HE image of resected brain metastasis.**

1. *Massive necrosis can be observed in the resected brain metastasis;* ***(B)*** *Dense tumor infiltrating lymphocytes. Original magnification: Panel A-B, hematoxylin and eosin, ×200.*
